# Impact of COVID-19 pandemic on mental health, social connectedness and cognitive performance of older adults

**DOI:** 10.1093/geroni/igab046.3497

**Published:** 2021-12-17

**Authors:** Juliana Souza-Talarico, Fernanda Silva, Maria Clara Jesus, Breno J A P Barbosa, Ricardo Nitrini, Sonia M D Brucki

**Affiliations:** 1 The University of Iowa, Iowa City, Iowa, United States; 2 School of Nursing, University of Sao Paulo, Sao Paulo, Sao Paulo, Brazil; 4 Faculty of Medicine, University of Sao Paulo, Sao Paulo, Sao Paulo, Brazil

## Abstract

The COVID-19 pandemic has profoundly impacted older adult's health and well-being worldwide. We explored the impact of the COVID-19 pandemic on daily activities and mental health and its relationship with cognitive performance in older adults. Methods: One-hundred individuals 60 years and older, without cognitive impairment and enrolled in the Brazilian Memory Study (BRAMS), a longitudinal study, were applied the UCLA Loneliness Scale, Perceived Stress Scale (PSS), Geriatric Depression Scale (GDS), and Mini-Mental State Examination (MMSE). Participants were asked whether they had changes in daily routine and social connectedness during the pandemic. Results: Almost half of the participants (48.4%) reported that the COVID-19 pandemic significantly affected their lives, 38.9% lost a relative or friend because of COVID-19, and 60% had daily routine changes. Relationships (40.5%) and emotion (22%) were reported as the most impacted area. Stopping physical activities and stay at home represented the main routine changed for 78% of participants. The use of voice messages through mobile phones to maintain social connectedness increased from 24.2% to 42.1%. For 38% of participants, their autonomy to daily decisions decreased, and 40% complained that memory got worse during the pandemic. More than 30% felt more stress, loneliness, or depression than in the pre-pandemic period. Controlling for age, sex, and education, higher loneliness scores were significantly associated with low MMES scores (p = 0.018). Conclusion: Significant changes in life, daily routine, social connectedness, and mental health-related to the COVID-19 pandemic were reported by older adult participants. Loneliness was associated with lower cognitive performance.

